# Obscure Skin Infection in a Morbidly Obese Patient: The Challenges of Diagnosis

**DOI:** 10.7759/cureus.92479

**Published:** 2025-09-16

**Authors:** Oscar Diaz, William J Morse, Noah M Krupnick, Lidiana Infante Sousa, Kevin Sande, Michel J Suarez, Lazaro Basart, Julio C Alvarez Hernandez

**Affiliations:** 1 Internal Medicine, Palmetto General Hospital, Hialeah, USA; 2 Internal Medicine, Dr. Kiran C. Patel College of Osteopathic Medicine, Nova Southeastern University, Fort Lauderdale, USA; 3 Medicine, Dr. Kiran C. Patel College of Osteopathic Medicine, Nova Southeastern University, Fort Lauderdale, USA; 4 Internal Medicine, Universidad de Ciencias Medicas de Holguin, Holguin, CUB

**Keywords:** lymphangitis, obesity, skin infections, soft tissue infections, thigh cellulitis

## Abstract

Obesity is a complex and increasingly prevalent condition associated with significant morbidity and mortality. Increased susceptibility to skin and soft tissue infections is attributable to multiple factors, including immune dysregulation, impaired mobility, and the anatomical challenges posed by excess adipose tissue. In morbidly obese individuals, limited access to intertriginous areas, difficulties with repositioning, and overlapping comorbidities can delay the recognition and treatment of infections, potentially worsening clinical outcomes. This case report describes a 50-year-old morbidly obese Cuban American male who presented to a hospital serving an underserved community with suspected cellulitis or lymphangitis of the lower extremity. No definitive source of infection was identified. Despite early empiric treatment, clinical deterioration ensued, resulting in respiratory failure and septic shock. The patient ultimately required tracheostomy and percutaneous endoscopic gastrostomy (PEG) tube placement, as well as long-term acute care. The case underscores the diagnostic challenges inherent in evaluating cutaneous and subcutaneous infections in patients with severe obesity. It highlights the importance of maintaining a high index of suspicion and employing a methodical approach to skin assessment in this population to ensure timely diagnosis and intervention in order to optimize clinical outcomes.

## Introduction

Obesity is a complex disease associated with significant morbidity and mortality. The prevalence of obesity is rising, and projections indicate that approximately 25% of the world's population will be affected by 2035 [1]. Obesity is a growing public health concern associated with a broad range of comorbidities, including an increased risk of skin and soft tissue infections [2,3]. The skin is particularly vulnerable in individuals with excess adipose tissue, which can contribute to impaired immune function, poor circulation, and mechanical stress within skin folds. These changes create an ideal environment for inflammation, moisture retention, and microbial overgrowth, predisposing patients to infections [4]. In morbidly obese individuals, early recognition of skin conditions can be particularly challenging due to limited access to intertriginous areas, difficulty repositioning patients, patient discomfort, and the potential for overlapping comorbidities to mask underlying conditions. The presence of a large pannus may further hinder early recognition and adversely impact outcomes.

This case report describes a morbidly obese Cuban American male who presented to our hospital serving an underserved community with a suspected skin infection of the lower extremity. It highlights the diagnostic challenges associated with skin examination in this patient population. It underscores the need for heightened clinical vigilance and a methodical approach to skin assessment in patients with obesity to ensure timely diagnosis and treatment of cutaneous infections.

## Case presentation

A 50-year-old Cuban American male with a history of morbid obesity, severe obstructive sleep apnea, pulmonary arterial hypertension, essential hypertension, moderate aortic stenosis, reduced ejection fraction heart failure, and chronic right lower extremity lymphedema presented to our hospital, which provides care to an underserved community, with increasing right leg pain and swelling. The swelling and lymphedema have progressed over the preceding five years. This has resulted in markedly decreased mobility, and the patient and family report that he is predominantly bedbound at this time. The patient describes his current pain as burning in nature, and it is associated with significant redness, warmth, and swelling. He denies any recent fever. The patient reports that the current symptoms have increased rapidly over the last three days.

**Figure 1:**
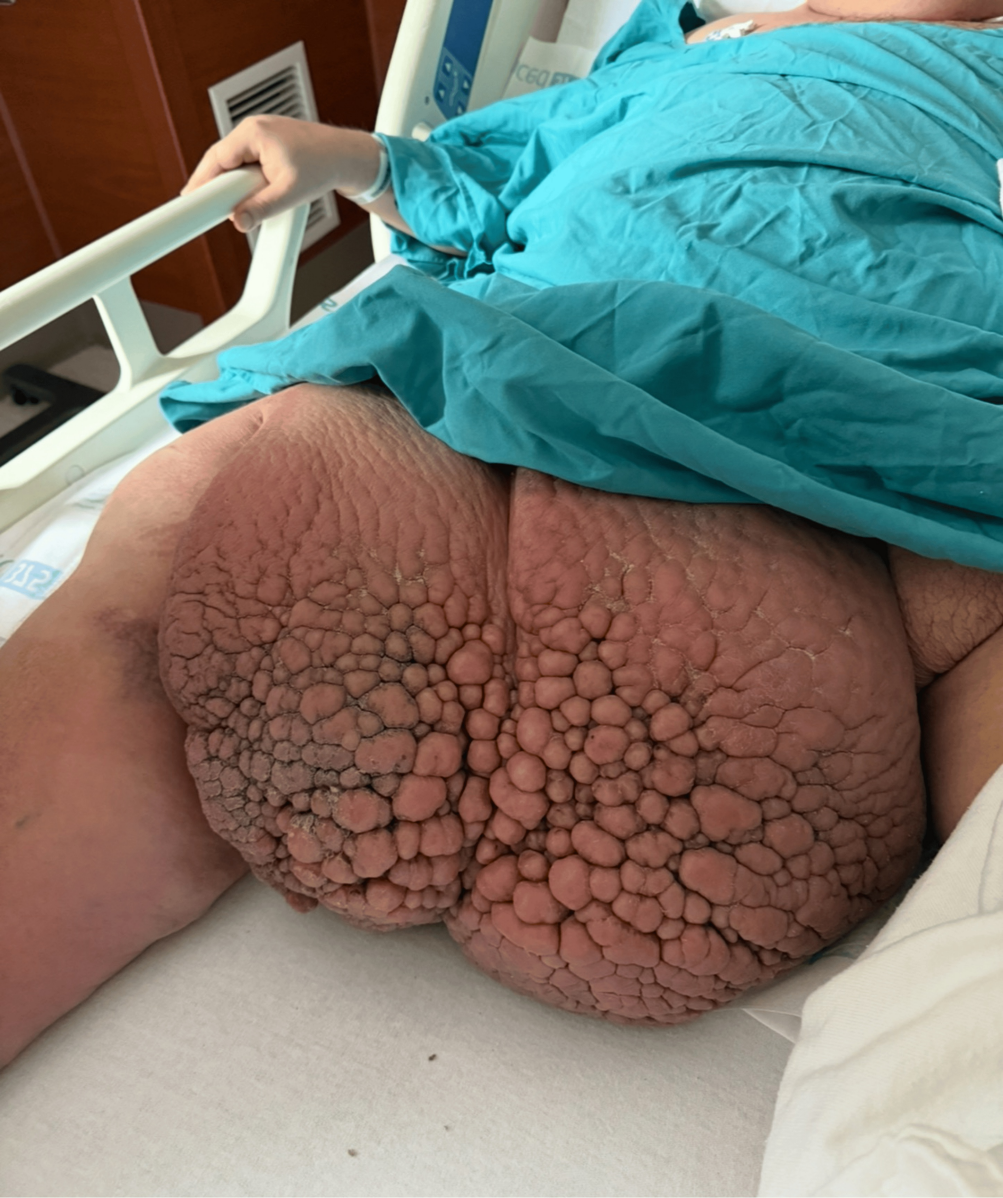
Right thigh pannus with papillomatosis

On initial evaluation, his vital signs were as follows: temperature, 99°F; blood pressure, 110/60 mmHg; pulse, 88 beats per minute; and respirations, 19 breaths per minute. Pertinent physical exam findings included that the patient was morbidly obese (BMI, 51.7 kg/m²). Examination of the chest revealed decreased breath sounds bilaterally, and a heart exam revealed distant heart sounds with an irregular rate. Examination of the extremities revealed markedly decreased range of motion, and the right lower extremity was erythematous, warm, and fluctuant. There was marked edema as well as areas of hyperkeratosis, papillomatosis, and pannus formation on the right thigh (Figure 1). There were areas of friability and excoriation within deep skin folds, and the pannus was markedly tender to palpation. There was no evidence of purulent drainage or bleeding.

Initial laboratory values are shown in Table 1. Ultrasound with Doppler in the lower extremities revealed no evidence of deep venous thrombosis.

**Table 1:** Initial laboratory values WBC: white blood cells; HGB: hemoglobin; HCT: hematocrit; PLT: platelets; PT: prothrombin time; INR: international normalized ratio; PTT: partial thromboplastin time; BUN: blood urea nitrogen; AST: aspartate aminotransferase; ALT: alanine aminotransferase; eGFR: estimated glomerular filtration rate

Test	Results	Normal Range
Complete Blood Count		
WBC	9.5 x 10^3^/uL	5.0–11.0 x 10^3^/uL
HGB	11.8 gm/dL	14.0–19.0 gm/dL
HCT	35.90%	42.0–52.0%
PLT	256 x 10^3^/uL	130–450 x 10^3^/uL
Coagulation Profile		
PT	11.4 seconds	9.4–12.5 seconds
INR (Calculated Value)	1	0.9–1.2
PTT	27.8 seconds	20.0–38.0 seconds
Complete Metabolic Panel		
Sodium	137 mmol/L	137–145 mmol/L
Potassium	3.8 mmol/L	3.4–5.0 mmol/L
Chloride	93 mmol/L	98–107 mmol/L
Carbon Dioxide	38 mmol/L	22–30 mmol/L
BUN	22 mg/dL	9.0–20.0 mg/dL
Creatinine	0.6 mg/dL	0.66–1.25 mg/dL
Glucose	99 mg/dL	74.0–106.0 mg/dL
AST	32 U/L	17–59 U/L
ALT	31 U/L	21–72 U/L
eGFR	118 mL/min/1.73 m^2^	≥ 90 mL/min/1.73 m^2^
Lactic Acid	0.9 mmol/L	0.7–2.0 mmol/L
Calcium	8.4 mg/dL	8.4–10.2 mg/dL
Total Bilirubin	0.70 mcg/dL	0.20–1.30 mcg/dL
Direct Bilirubin	0.0 mg/dL	0.0–0.3 mg/dL
Indirect Bilirubin	0.7 mg/dL	0.0–0.1 mg/dL
Alkaline Phosphatase	86 U/L	38–126 U/L

**Figure 2:**
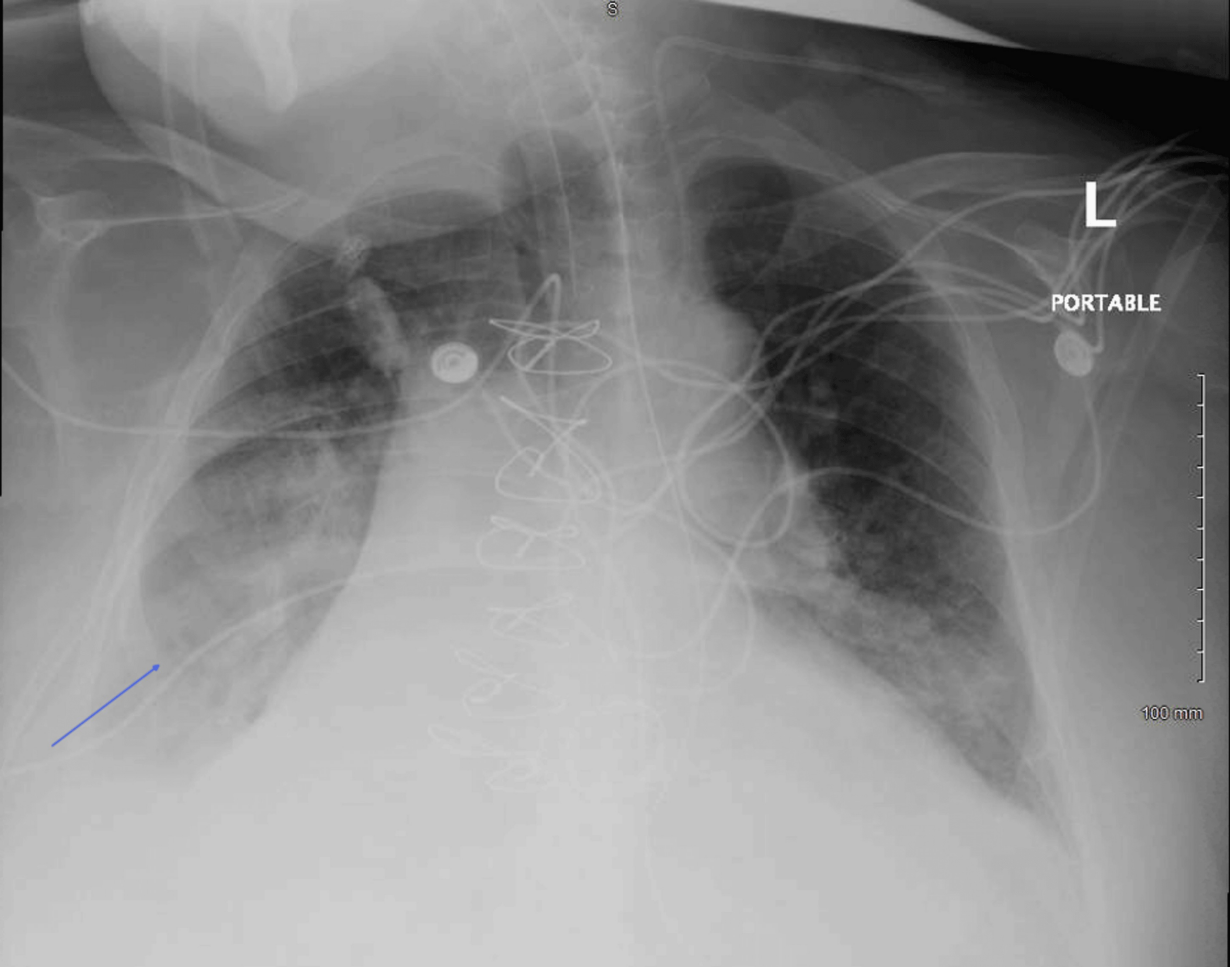
Portable chest X-ray showing right basilar infiltrate

He was admitted and treated with a 10-day course of vancomycin and ceftriaxone for suspected cellulitis and lymphangitis after blood cultures were taken. Given the absence of definitive signs of infection, the antibiotics were replaced with topical nystatin on hospital day three. Several hours later, the patient developed increasing shortness of breath, respiratory insufficiency, and hypotension that required intubation, mechanical ventilation, and vasopressor support. A CT angiogram revealed no pulmonary embolus. The chest X-ray was of limited quality due to the patient's body habitus but showed a right basilar infiltrate, raising concern for aspiration pneumonia (Figure 2). At this time, the patient was found to be febrile to 102.9℉. Empiric treatment for septic shock with piperacillin/tazobactam was initiated. Initial blood cultures grew *Staphylococcus caprae*, but all subsequent cultures following the initiation of antibiotics showed no evidence of bacterial growth. A transesophageal echocardiogram, performed to exclude endocarditis, revealed no evidence of vegetations. Wound cultures grew *Proteus* and *Citrobacter* species; however, after consultation with the infectious disease team, these findings were attributed to skin colonization rather than active infection.

The patient remained intermittently febrile, which caused persistent concern for underlying skin, soft tissue, or pulmonary infection. His temperature curve gradually improved over the next week, and antibiotics were discontinued. The patient underwent several days of unsuccessful weaning trials. He subsequently required tracheostomy and percutaneous endoscopic gastrostomy (PEG) tube placement and will require long-term acute care.

## Discussion

Obesity is a major global health concern associated with significant morbidity and mortality. According to the World Health Organization, obesity is defined as excessive fat accumulation that presents a risk to health, with a BMI ≥ 30 kg/m² classified as obese and a BMI ≥ 40 kg/m² considered severely obese. Since 1980, the global prevalence of obesity has approximately doubled, with rising rates observed across all age and socioeconomic groups and both sexes [5]. In the United States, approximately two in five adults are obese, and one in 10 are severely obese [6]. Obesity is a chronic inflammatory condition [1]. Affected individuals are at increased risk of developing multiple diseases, including atherosclerosis, diabetes mellitus type 2, and cancer [2]. It is also associated with an increased risk of infection in both genders [3]. Globally, obesity contributes to more than five million deaths each year [1], underscoring its profound impact on public health.

The skin is the largest organ in the human body, and 50% of individuals with obesity exhibit skin changes. These include hypertrophic conditions such as acanthosis nigricans and infections [2]. Lymphedema with morbid obesity is associated with a greater than two-fold increase in cellulitis [7]. Proposed mechanisms for the increased incidence of skin and subcutaneous infections in obese individuals include that adipose tissue exerts immunosuppressive effects through adipokines and that skin folds impede blood flow and increase the likelihood of abscesses and infections [8]. Morbidly obese individuals face unique challenges related to skin health, such as impaired hygiene, poor mobility, and skin folds that cause irritation, chafing, and moisture trapping, which creates an ideal environment for the growth of bacteria and fungi. This places them at increased risk for bacterial and *Candida* infections [4]. Intertrigo, an inflammatory condition caused by friction in warm, moist areas, is also associated with obesity [2,7]. Additionally, pannus, characterized by excess skin and subcutaneous tissue, can become a nidus for chronic cellulitis, rashes, ulcers, and ischemic panniculitis [9].

This case underscores the importance of maintaining a high index of suspicion for skin and subcutaneous tissue-related infections in patients with obesity, highlighting the clinical challenges of conducting a thorough physical examination of the skin in this population. Excess adipose tissue can obscure signs of infection, including erythema, warmth, or fluctuance, leading to missed or delayed diagnoses. In addition, moisture, friction, and poor ventilation in skin folds further contribute to skin breakdown and secondary infection.

Infection was initially suspected in our patient, who is morbidly obese with limited mobility, impaired hygiene, and deep skin folds. He presented with a large, erythematous, and tender pannus on his lower leg, raising concern for cellulitis or lymphangitis. In obese patients, comprehensive skin assessment is often hindered by limited access to intertriginous areas and the need to lift or reposition large skin folds, which can be painful, as it was in this case. Another obstacle is the risk of exacerbating comorbidities such as respiratory or cardiovascular compromise during repositioning. This case emphasizes the need for a careful, methodical approach to skin examination in patients with obesity, including the use of adjunct lighting, assistance with repositioning, and attention to subtle clinical cues to ensure early detection and appropriate management of cutaneous infections. The patient's body habitus posed significant challenges in identifying a definitive infectious source for his sepsis, resulting in delayed initiation of targeted treatment. This delay, combined with obesity-related pulmonary comorbidities, likely contributed to the eventual need for tracheostomy and PEG tube placement. Had these obstacles not been present, earlier identification and management of the infection may have led to a more favorable clinical outcome.

## Conclusions

This case highlights the critical need for heightened clinical vigilance and adapted examination techniques when evaluating obese patients, particularly those with limited mobility and impaired hygiene. The diagnostic challenges presented by excess adipose tissue, deep skin folds, and comorbidities can delay the recognition and treatment of cutaneous infections, potentially resulting in life-threatening complications such as sepsis. In this patient, the inability to promptly identify the source of infection, combined with significant pulmonary comorbidities, contributed to a prolonged hospital course and the need for long-term supportive care.

Healthcare providers must be equipped with the tools and awareness to meet the complex needs of obese patients. A methodical, multidisciplinary approach to skin assessment, including physical examination support, enhanced lighting, and consideration of nontraditional infectious sources, is essential. Early and accurate diagnosis not only improves individual patient outcomes but also helps reduce healthcare costs and resource utilization associated with preventable complications in obesity-related infections.
